# The Role of Mammalian Glial Cells in Circadian Rhythm Regulation

**DOI:** 10.1155/2017/8140737

**Published:** 2017-12-25

**Authors:** Donají Chi-Castañeda, Arturo Ortega

**Affiliations:** ^1^Laboratorio de Neurotoxicología, Departamento de Toxicología, Centro de Investigación y de Estudios Avanzados del Instituto Politécnico Nacional, Apartado Postal 14-740, 07000 Ciudad de México, Mexico; ^2^Soluciones para un México Verde S.A. de C.V., 01210 Ciudad de México, Mexico

## Abstract

Circadian rhythms are biological oscillations with a period of about 24 hours. These rhythms are maintained by an innate genetically determined time-keeping system called the circadian clock. A large number of the proteins involved in the regulation of this clock are transcription factors controlling rhythmic transcription of *so-called* clock-controlled genes, which participate in a plethora of physiological functions in the organism. In the brain, several areas, besides the suprachiasmatic nucleus, harbor functional clocks characterized by a well-defined time pattern of clock gene expression. This expression rhythm is not restricted to neurons but is also present in glia, suggesting that these cells are involved in circadian rhythmicity. However, only certain glial cells fulfill the criteria to be called glial clocks, namely, to display molecular oscillators based on the canonical clock protein PERIOD, which depends on the suprachiasmatic nucleus for their synchronization. In this contribution, we summarize the current information about activity of the clock genes in glial cells, their potential role as oscillators as well as clinical implications.

## 1. Introduction

Most light-sensitive organisms have built-in time-measuring devices that are commonly known as circadian clocks. The term *circadian* was introduced by Halberg to describe the biological rhythms that have a period of approximately 24 h and are known as circadian rhythms [1]. Circadian rhythms are present along the phylogenetic scale, in mammals regulate a plethora of functions such as the rest-activity cycle, hormone secretion, and daily variations in metabolism and body temperature [2].

The intracellular circadian clock is based on a transcription-translation feedback system that drive the self-sustaining clock mechanism in the suprachiasmatic nucleus (SCN, the “master clock”) and in peripheral tissues (“peripheral clocks”) [3, 4]. At the molecular level, the molecular machinery that generates circadian rhythms involves CLOCK- (circadian locomotor output cycles kaput) BMAL1 (brain and muscle aryl hydrocarbon receptor nuclear translocator-like protein 1) heterodimers that control the periodic expression of *Per* (*periods 1–3*) and *Cry* (*cryptochrome 1,2*) genes. These gene products form the PER-CRY heterodimer that is translocated to the nucleus inhibiting their own transcription. Additionally, an accessory regulatory loop involves the rhythmic regulation of *Bmal1* transcription through the coordinated action of the transcriptional repressor *REV-ERBα* (*Reverse Erb alpha*) and the transcriptional activator *RORα* (*retinoid-related orphan receptor-alpha*) [5–8].

In mammals, the SCN synchronizes multiple peripheral clocks, in numerous tissues and cell types, presumably via the combination of neural and humoral signaling [2, 9]. The general consensus of the cellular identity of the oscillating cells in the brain points to the neurons, although it has been demonstrated that the glial cells are circadian oscillators as well, and their synchronization also depends on the SCN [10–12].

Glial cells make up a large fraction of human nervous system cells, with numbers exceeding those of neurons by a factor of ten, depending on the brain structure studied. Particularly, glial cells occupy about half the volume of the brain and participate in diverse functions, including regulation of synaptic transmission, plasticity, behavior, and synapse development, and these cells are also involved in neurodegeneration [13–17]. Interestingly, it has been described that glial cells also play an important role in the regulation of circadian rhythms [18–23], although little attention has been paid to this function. Accordingly, we summarize here the recent findings about clock genes in glial cells, the plausible role of the glial cells as cellular oscillators, and the possible medical implications of clock genes in this cell type.

## 2. Clock Genes in Glial Cells

### 2.1. Astrocytes

This type of glial cell is involved in the buffering of extracellular K^+^, regulating neurotransmitter release [24], forming the blood-brain barrier, releasing growth factors, and the regulation of complex brain mechanisms, such as sleep homeostasis [25] and memory [26–28], among other functions.

In 1990, it was suggested that glial cells might express molecular oscillators, which are based on the clock protein PER. Particularly, it was demonstrated that PER was localized both in neurons and glial cells of the fly brain, which showed robust circadian rhythms and abundance [11]. Subsequently, Ewer and coworkers reported that certain weakly rhythmic flies contained detectable PER only in glia, suggesting that glial oscillators play an important role in the pacemaker driving rhythmic behavior [12]. Later, it was confirmed the rhythmic expression of clock genes in rat and mouse astrocytes, indicating that these cells contain a PER-based molecular oscillator that damps in the absence of neuronal signals [29, 30]. These astroglial cultures were capable to display a sustained rhythmicity for 7 days when cocultured with SCN explants, whereas cortical explants did not influence rhythmicity [29], suggesting that the presence of sustained rhythms in glial cells requires a secreted neuronal factor expressed in the SCN. Temperature cycles entrain *Per1* rhythms in astroglial cultures [29] however are unlikely to be a relevant factor, since exposure to SCN explants sustained glial rhythms without any change in temperature.

Several studies have explored the role of the mammalian PER-based oscillator in glial physiology. It has been reported that *Glast* (*glutamate/aspartate transporter*) expression and protein levels within the SCN present a diurnal rhythm in a light/dark (12/12 h) cycle [31]. However, it was not determined whether this rhythm persist or not in nonrhythmic conditions (constant darkness or constant light), in other words in lack of environmental information. Results of Spanagel and collaborators are complementary with the observation concerning to GLAST levels, which do not display an obvious rhythmicity in the *Per2* mutant mice pointing out the presence of a circadian control [31]. Beaulé and colleagues reported that cultured cortical astrocytes from *Clock* mutant animals have reduced *Glast* mRNA and protein levels [32], proposing that the vast majority of glial glutamate (Glu) uptake activity is a function of the transcription factors *Clock* and *Npas2* (*neuronal PAS domain protein 2*) and of the transcriptional regulator *Per2* [32, 33]. This dependence could be explained by the involvement of CLOCK and NPAS2 in the indirect regulation of *Glast* transcription or in GLAST protein stabilization and/or localization [34]. It should be noted that no evidence has been demonstrated for circadian changes in Glu uptake, suggesting a noncircadian role for clock proteins that might be involved in the regulation of *Glast* gene transcription or *Glast* mRNA translation and/or stability [32, 33].

Concerning Glu, it is known that this neurotransmitter participates in photic entrainment of circadian rhythms. In 2015, it was reported that in cultured Bergmann glial cells, BMAL1 expression is Glu time- and dose-dependent. This phenomena might be a result of stabilization of the protein after it has been phosphorylated by PKA (cyclic AMP-dependent protein kinase) and/or PKC (Ca^2+^/diacylglycerol-dependent protein kinase), pointing out that Glu is critically involved in glial BMAL1 expression and that glial cells are important in the control of circadian rhythms in the cerebellum [22].

It has been recently demonstrated that not only SCN neurons but also SCN astrocytes possess pacemaking properties [35]. By using long-term live imaging, Brancaccio and colleagues simultaneously codetected circadian oscillations of neuronal and astrocytic intracellular calcium ([Ca^2+^]_**i**_) within the SCN, with [Ca^2+^]_**i**_ levels peaking during the circadian day and night. Thereby, these oscillations of [Ca^2+^]_**i**_ were antiphasic and showed a complementary waveform [35]. In the same study, it was reported widespread circadian oscillations of extracellular Glu ([Glu]_**e**_) in the SCN in phase with astrocytic [Ca^2+^]_**i**_. These circadian oscillations of [Glu]_**e**_ are generated intrinsically in the SCN and also depend directly on astrocytic metabolism [35]. Using pharmacological inhibition of the glial and neuronal isoforms of the Glu transporters, a continuing circadian oscillation of Glu release by astrocytes is observed. Remarkably, [Glu]_**e**_ oscillations are generated by concerted rhythms of release and uptake, and blocking Glu uptake impairs the fine-tuning of the [Glu]_**e**_/[Ca^2+^]_**i**_ relationship, reducing the robustness of the rhythms of neuronal [Ca^2+^]_**i**_ across the SCN. Consequently, SCN cellular oscillators progressively desynchronize, until the [Glu]_**e**_/[Ca^2+^]_**i**_ alignment is restored. Presynaptic NMDA (N-methyl-D-aspartate) receptors 2C-mediated glutamatergic gliotransmission inhibit neuronal activity during circadian night, and this mechanism is essential to sustain circadian rhythmicity in the dorsal SCN [35]. Accordingly, during the circadian night, SCN astrocytes are metabolically active (high [Ca^2+^]_**i**_) and release high levels (baseline activity) of Glu into the extracellular space, which in turn activates presynaptic NR2C-expressing neurons in the dorsal SCN, thereby increasing GABAergic inhibitory tone across the circuit. In contrast, during the circadian day, [Glu]_**e**_ is reduced by diminished glial release and increased EAAT-mediated Glu uptake and consequently, GABAergic tone is reduced, thereby derepressing spontaneous membrane potential, neuronal [Ca^2+^]_**i**_, and facilitating electrical firing [35].

Moreover, it was reported that SCN astrocytes are functional circadian oscillators, which modulate the period of SCN and the rest-activity rhythms [36]. The loss of rhythm in SCN astrocytes by *Bmal1* deletion leads to an extended circadian period of rest-activity rhythms. This *Bmal1* deletion in a small proportion of SCN cells appears to change the period of the SCN and behavior by the loss of rhythmicity in 20% of SCN cells that express AVP (arginine-vasopressin) or 10% of cells that express Aldh1L1 (specific astrocytic marker) or GFAP (glial fibrillary acid protein) [36].

Earlier studies in SCN astrocytes revealed high-amplitude daily rhythms in the expression of GFAP [37] and their coverage of the soma and dendrites of vasointestinal polypeptide- and AVP-expressing SCN neurons, which are related with modifications in synaptic innervation of these neurons [38, 39]. Rhythmic pattern of GFAP was observed in constant darkness in the SCN of hamsters, rats, and mice [37, 40], suggesting that these rhythms are intrinsic and independent of external light cues. Although the role of daily oscillations of GFAP in SCN is unknown, it has been associated with two main aspects of the clock functioning: metabolic exchanges and plasticity [38]. According to this last aspect, it has been demonstrated that mice lacking the *Gfap* show impaired long-term depression in the cerebellum, reduced eye-blink conditioning [41], longer periods of activity, and more arrhythmicity in constant light conditions compared to wild type [40, 42]. These results indicate that GFAP in glial cells plays a role in the regulation of neuronal function.

A daily variation of GFAP in the mouse SCN, as well as the NF-*κ*B (nuclear factor-*κ*B) expression in SCN astrocytes has been documented using tissue slices and primary cell cultures [43]. Particularly, in the latter case, LPS (lipopolysaccharide), IL-1*α* (interleukin-1 alpha), and TNF*α* (tumor necrosis factor alpha) promoted the activation of NF-*κ*B, indicating that SCN astrocytes mediate the input signals to the circadian system from the immune system via NF-*κ*B signaling [43].

ROR*α* is expressed in astrocytes but not in microglia. Studies using *staggerer* mice, which have a 122 bp deletion in the *RORα* gene, allowed the identification of several functions of this nuclear receptor, both in the periphery and in the CNS. Interestingly, a massive cerebellar neurodegeneration leading to severe ataxia was also observed in these mice [44].

Furthermore, it has also been reported that *RORα*-deficient mice have abnormal immune responses, associated with increased levels of IL-1*β*, IL-6, and TNF*α* [45]. An additional report showed that in primary astrocyte cultures, ROR*α* directly participates in the regulation of the inflammatory reaction via the inhibition of the NF-*κ*B pathway. Thus, in a noninflammatory condition, the nuclear receptor directly increases IL-6 expression, while in an inflammatory condition, ROR*α* reduces cytokine-induced *Il-6* upregulation [46].

Other nuclear receptors involved in the inflammatory response are REV-ERB*α* and REV-ERB*β*; both receptors are expressed in rat C6 cells and in astrocyte cultures derived from rat cortex and spinal cord [47]. Particularly, in rat C6 astroglial cells, it has been reported that TNF significantly increases chemokine *Ccl-2* (*monocyte chemoattractant protein-1*), *Il-6*, *iNOS* (*inducible nitric oxide synthase*), and *Mmp-9* (*matrix metalloprotease-9*) mRNA levels. However, both isoforms of REV-ERB inhibit TNF-induced upregulation of *Ccl-2* and *Mmp-9* mRNA levels. Particularly, REV-ERB*α* and REV-ERB*β* decrease MMP-9 expression via HDAC3 (histone deacetylase 3) [47]. Moreover, it has been shown that REV-ERB*α* inhibits *Il-6* upregulation in murine skeletal muscle cells and macrophages [48–50], suggesting that the activity of this nuclear receptor is tissue-specific.

Circadian expression of clock genes such as *Per1*, *Per2*, *Cry1*, and *Bmal1* can be observed in mice spinal cord. Surprisingly, circadian expression of *GS* (*glutamine synthetase*, a glial-enriched enzyme) and *COX*-*1* (*cyclooxygenase-1*) at both mRNA and protein levels was also detected in the same brain area [51]. Moreover, circadian changes in the expression of GS suggest that astrocyte metabolism is subjected to circadian modulation. Whereas, the disruption of astroglial function using fluorocitrate (a glial metabolic inhibitor) led to the suppression of the oscillating expression of not only GS and COX-1 but also the expression of clock genes. These findings suggest that spinal circadian expression of clock genes depends on the activity of astrocytes, since the inhibition of astrocytic function disrupts circadian gene mRNA expression [51].

Gliotransmission is the process by which astrocytes communicate with immediate glia and neurons through the release of transmitters such as ATP and Glu [42, 52, 53]. *In vivo*, a circadian pattern of ATP release appears to derive primarily from astrocytes within the SCN; however, the functional implications of these extracellular ATP rhythms are unknown [54]. Moreover, it has been shown that astrocytes display daily extracellular ATP oscillations that rely on key clock genes (*Clock*, *Per1*, and *Per2*) and inositol triphosphate signaling [55], suggesting that extracellular ATP levels are augmented at specific hours of the day, and probably, a clock-induced increase in energy metabolism and glial activity is present [55].

Mammalian and insect glial cells modulate circadian neuronal circuitry and behavior via glial calcium signaling [19]. Genetic manipulations of glial vesicle trafficking, the membrane ionic gradient, or internal calcium storage all lead to arrhythmic locomotor activity in *Drosophila*, an organism in which astrocytes, but not other glial cell types, are relevant for the circadian modulation of behavior. It should be noted that *Drosophila* and mammalian astrocytes elicit similar functions due to their preserved morphology and molecular signatures. Besides, PER-based glial oscillator is not essential for the free-running behavioral rhythmicity, although the possibility that this oscillator is required for circadian photic sensitivity or the expression of a different rhythm cannot be ruled out [19].

Recently, Xu and colleagues reported the existence of canonical circadian clock genes in mammalian retinal Müller glia. This study not only demonstrated that retinal Müller cells generate molecular circadian rhythms isolated from other retinal cell types but also demonstrated that these retinal cells exhibit unique features of their molecular circadian clock compared to the retina as a complex system. However, it is important to highlight that the authors mention that both mouse and human Müller cells exhibit species-specific differences in the gene dependence of their clocks [56]. Accordingly, it was observed that human Müller cells exhibit *in vitro* circadian rhythms in clock gene expression, although the rhythm in these cells does not seem to depend on *Per1* expression. Whereas, in mouse Müller cells, knockout or knockdown of *Per1* led to arrhythmicity, suggesting that human Müller cells may have a decreased dependence in *Per1* expression to regulate rhythmicity [56]. Additional evidence reported by Tosini and Menaker demonstrates that the mammalian neural retina contains a genetically programmed circadian oscillator [57]. Nevertheless, Xu and coworkers propose to Müller glia as a candidate clock cell population in the mammalian retina [56]. The results obtained in both reports indicate that both neurons and glia play an important role in the generation of circadian rhythms in this autonomous oscillator.

### 2.2. Microglia

These glial cells are the main innate immune cells of the CNS and play essential functions in the maintenance of neuronal circuitry, regulation of behavior, and functional state of neurotransmission [20, 21].

Knowledge about a molecular clock in this type of glial cells is relatively recent. In 2011, it was demonstrated that the clock genes are constitutively expressed in both cultured murine microglia and the microglial cell line BV-2 cells. In the same study, it was also reported that ATP selectively promotes the expression of mRNA and corresponding protein for *Per1* via P2X7 purinergic receptor subtype in microglial cells [58]. Years later, it was confirmed that cortical microglia contain an intrinsic molecular clock capable of regulating diurnal changes of its morphological aspect [20]. Specifically, it has been demonstrated in mice that microglia controls the sleep-wake cycle-dependent changes in synaptic strength through the extension and retraction of their processes [21]. Hayashi and colleagues showed that CatS (Cathepsin S, a microglia-specific lysosomal cysteine protease in the brain) exhibits a circadian expression in cortical microglia. Such expression of CatS induces diurnal variations in the synaptic strength of the cortical neurons via the proteolytic modification of the perineuronal environment. Conversely, alterations in CatS lead to hyperlocomotor activity, as well as the deletion of the diurnal variations in the synaptic activity and dendritic spine density of the cortical neurons as a consequence of failure to downscale the synaptic strength during sleep [20, 59]. This process is necessary for the acquisition of subsequent novel information after waking [20]; therefore, dysfunction of microglia intrinsic circadian clock could be involved in social behavior abnormalities [59] and neuropsychiatric disorders, including depression and cognitive impairment [60, 61].

In 2015, Fonken and coworkers reported that microglia possesses circadian clock mechanisms and displays rhythmic fluctuations in both basal inflammatory gene expression and inflammatory potential. It is interesting to note that inflammatory potential in microglia is associated with time-of-day differences, this is because of the circadian differences observed in sickness response [23].

Recently, Nakazato and colleagues demonstrated that *Bmal1* modulates *Il-6* upregulation in microglial cells exposed to LPS using siRNA targeting *Bmal1* and *Bmal1*-deficient mice [62]. These results suggest that an intrinsic microglial clock may regulate microglial inflammatory responses under pathological conditions *in vivo*. It was also observed that *Bmal1* bindings to the *Il-6* promoter region only in cells exposed to LPS; for which, they suggest that histone modification occurred at the *Il-6* promoter region with E-box elements [62].

### 2.3. Oligodendrocytes

These cells are the myelinating glia of the CNS, provide axonal metabolic support [63], and contribute to neuroplasticity [64]. Scarce information regarding clock genes in these cells is available. A previous study suggested that oligodendrocytes' proliferation depends on the time-of-the-day in the hippocampal *hilus*, indicating a close connection between the temporal information and glial cells in this structure [65]. To date, there is no report showing that oligodendrocytes have an internal circadian clock. However, it has been suggested that clock genes might regulate OPC (oligodendrocyte precursor cell) proliferation, since these cells in the hippocampus express cyclin D1 [18], which is regulated by *Per2* gene [66].

## 3. Clinical Implications

Recent studies indicate that defective clock genes in glial cells participate in diverse brain pathologies, mainly in psychiatric diseases. However, it is important to keep in mind that a single clock gene can have different repercussions on health and that several clock genes may be related to the same pathology (for detailed review, see reference [67]). Particularly, mutations in *Clock*, *Npas2*, and/or *Per2* are all involved in a hyperglutamatergic scenario due to a decrease in GLAST expression and as consequence, a reduction in Glu uptake [31, 32, 68]. In this scenario, astrocytic Glu release has clear pathophysiological implications like stroke, multiple sclerosis, and dementia [69]. Additionally, it has been established that Glu regulates the levels of dopamine and other neurotransmitters and neuropeptides that mediate both positive and negative aspects of drug reinforcement and reward. In this manner, both hyper- and hypoglutamatergic states in specific brain areas are directly involved in different stages of addiction, including development, persistence, and abstinence [68]. Fascinatingly, clock genes participate in the modulation of common mechanisms of drug abuse-related behaviors [31, 70].

Moreover, alterations in *Per1*, *Per3*, and *Bmal1* lead essentially to changes in both short- and long-term memory, chronic oxidative stress in the brain, variations in cocaine sensitization, and association with a number of psychiatric diseases [71–77]. Similarly, *Npas2*, *Gsk3β*, *Dbp*, *Cry1*, and *Clock* are involved in variations in drugs sensitization, as well as in diverse psychiatric diseases, mainly bipolar disorder, schizophrenia, Alzheimer, and unipolar major depressive disorder [32, 78–82].

Nowadays, disturbances in the sleep parameters are common. These disturbances are associated with a spectrum of neurological and psychiatric disorders. Interestingly, clock genes are also involved in variations related with sleep time, sleep fragmentation, and atypical responses following sleep deprivation [83–85]. However, sleep disruptions also have severe consequences in the immune system, leading to an impaired immune function [86, 87]. In line with these reports, it has been established that immune cells exhibit circadian expression of clock genes, which in turn, participate in the regulation of diverse immunological activities. Particularly, *REV-ERB* is involved in neurodegenerative disorders with an inflammatory component [47]. It has been demonstrated that this clock gene represses macrophage gene expression [88] and targets inflammatory function of macrophages through the direct regulation of *Ccl-2* [50]. On the other hand, *Bmal1* controls rhythmic trafficking of inflammatory monocytes to sites of inflammation [89]. Taking these reports together, it is possible to suggest that circadian disruptions exacerbate inflammatory responses in both periphery [90] and CNS [91].

Additionally, it has been shown that *RORα* is an important molecular player in diverse pathological processes including oxidative stress-induced apoptosis and cerebral hypoxia, both in neurons and astrocytes, due to its neuroprotective properties [44].

Finally, abnormal microglial cells are also associated with neurological disorders [92–94]. Taking into consideration that a risk factor for psychiatric diseases is the dysfunction of the clock system, it is relevant to suggest that the microglial clock might be an interesting target for the development of novel neurological therapeutic agents.

## 4. Conclusion

The expression of clock genes in glial cells has great importance for the maintenance of a healthy brain (Table 1). Actually, clock genes are relevant for the development of novel strategies for the treatment of a wide range of human diseases such as metabolic and cardiovascular diseases, immune system dysfunction, neuropsychiatric disorders, and even cancer. Specifically, changes in the expression of clock genes in glial cells lead to problems related to an imbalance of the glutamatergic system, resulting in neurological disorders; therefore, understanding the role that glial cells play in brain circadian physiology is extremely relevant.

## Figures and Tables

**Table 1:** Circadian functions regulated by the glial cells.

CG/CCG/molecule	Circadian functions	References
*Astrocytes*		
*Clock*	Regulation of the glutamatergic system (*Glast* mRNA and protein levels)	[32]
	Modulates ATP release	[55]
*Npas2*	Regulation of the glutamatergic system (*Glast* mRNA and protein levels)	[32]
*Per1*	Regulation of nociceptive processes	[51]
	Modulates ATP release	[55]
*Per2*	Regulation of the glutamatergic system (GLAST protein levels)	[31, 32]
	Regulation of nociceptive processes	[51]
	Modulates ATP release	[55]
	Regulates to *cyclin D1*	[66]
*Bmal1*	Modulates the period of the SCN and behavior	[36]
	Regulation of nociceptive processes	[51]
*Cry1*	Regulation of nociceptive processes	[51]
*Gfap*	Participates in metabolic exchanges and plasticity	[38, 40–42]
NF-*κ*B	SCN astrocytes mediate the immune signals to the circadian system via NF-*κ*B signaling	[43]
*RORα*	Participates in the regulation of the inflammatory response (inhibits NF-*κ*B pathway and regulates IL-6 expression)	[44–46]
*REV-ERBα/REV-ERB β*	Participates in the regulation of the inflammatory response (both isoforms inhibit TNF-induced upregulation of *Ccl-2* and *Mmp-9*; and *REV-ERBα* inhibits *Il-6* upregulation)	[47–50]
*GS*	Regulation of the glutamatergic system (glutamate-glutamine metabolic cycle)	[51]
	Regulation of various spinal sensory functions	[51]
*COX-1*	Regulation of various spinal sensory functions	[51]
IP_3_	Modulates ATP release (IP_3_-dependent calcium signaling)	[55]
ATP	Regulation of the energy metabolism and glial activity	[55]
Ca^2+^	Modulation of circadian behavior	[19]
	Regulates the release of gliotransmitters	[35]
Glu	Regulates BMAL1 expression (Glu time- and dose-dependent)	[22]
	Provides the inhibitory astrocytic-neuronal coupling signal during nighttime in the SCN via NMDAR2C	[35]

*Microglia*		
ATP	Upregulates the *Per1* mRNA expression via P2X7 purinergic receptor subtype	[58]
CatS	Regulates the synaptic strength, including neuronal transmission and spine density via the proteolytic modification of the perineuronal environment	[20, 21, 59]
*Bmal1*	Implicated in the inflammatory response (modulates *Il-6* upregulation)	[62]

*Oligodendrocytes*		
*Cyclin D1*	Regulation of the OPC proliferation	[18]

ATP: adenosine triphosphate; *Bmal1*: *brain and muscle ARNT-like protein 1*; Ca^2+^: calcium; CatS: cathepsin S; CGs: clock genes; CCGs: clock-controlled genes; *Ccl-2*: *monocyte chemoattractant protein-1*; *Clock*: *circadian locomotor output cycles kaput*; *COX-1*: *cyclooxygenase-1*; *Cry1*: *cryptochrome 1*; *Gfap*: *glial fibrillary acidic protein*; GLAST: glutamate aspartate transporter; Glu: glutamate; *GS*: *glutamine synthetase*; *Il-6*: *interleukin-6*; IP_3_: inositol triphosphate; *Mmp-9*: *matrix metalloprotease-9*; NF-*κ*B: nuclear factor-kappaB; NMDAR2C: N-methyl-D-aspartate receptor 2C subunit; *Npas2*: *neuronal PAS domain protein 2*; P2X7: purinoreceptor; OPCs: oligodendrocyte precursor cells; *Per*: *period*; *REV-ERB*: *reverse Erb*; *RORα*: *retinoid-related orphan receptor-alpha*; SCN: suprachiasmatic nucleus; TNF: tumor necrosis factor.

## References

[1] Halberg F. (1959). Physiologic 24-hour periodicity; general and procedural considerations with reference to the adrenal cycle. *Internationale Zeitschrift fur Vitaminforschung Beiheft*.

[2] Stratmann M., Schibler U. (2006). Properties, entrainment, and physiological functions of mammalian peripheral oscillators. *Journal of Biological Rhythms*.

[3] Reppert S. M., Weaver D. R. (2002). Coordination of circadian timing in mammals. *Nature*.

[4] Lowrey P. L., Takahashi J. S. (2004). Mammalian circadian biology: elucidating genome-wide levels of temporal organization. *Annual Review of Genomics and Human Genetics*.

[5] Dunlap J. C. (1999). Molecular bases for circadian clocks. *Cell*.

[6] Harmer S. L., Panda S., Kay S. A. (2001). Molecular bases of circadian rhythms. *Annual Review of Cell and Developmental Biology*.

[7] Reppert S. M., Weaver D. R. (2001). Molecular analysis of mammalian circadian rhythms. *Annual Review of Physiology*.

[8] Preitner N., Damiola F., Luis-Lopez-Molina (2002). The orphan nuclear receptor REV-ERBα controls circadian transcription within the positive limb of the mammalian circadian oscillator. *Cell*.

[9] Schibler U., Sassone-Corsi P. (2002). A web of circadian pacemakers. *Cell*.

[10] Siwicki K. K., Eastman C., Petersen G., Rosbash M., Hall J. C. (1988). Antibodies to the period gene product of Drosophila reveal diverse tissue distribution and rhythmic changes in the visual system. *Neuron*.

[11] Zerr D. M., Hall J. C., Rosbash M., Siwicki K. K. (1990). Circadian fluctuations of period protein immunoreactivity in the CNS and the visual system of Drosophila. *The Journal of Neuroscience*.

[12] Ewer J., Frisch B., Hamblen-Coyle M. J., Rosbash M., Hall J. C. (1992). Expression of the period clock gene within different cell types in the brain of Drosophila adults and mosaic analysis of these cells’ influence on circadian behavioral rhythms. *The Journal of Neuroscience*.

[13] Jessen K. R., Richardson W. D. (2001). *Glial Cell Development: Basic Principles and Clinical Relevance*.

[14] Jessen K. R. (2004). Glial cells. *International Journal of Biochemistry and Cell Biology*.

[15] Stork T., Bernardos R., Freeman M. R. (2012). Analysis of glial cell development and function in Drosophila. *Cold Spring Harbor Protocols*.

[16] Clarke L. E., Barres B. A. (2013). Emerging roles of astrocytes in neural circuit development. *Nature Reviews Neuroscience*.

[17] Brown G. C., Neher J. J. (2014). Microglial phagocytosis of live neurons. *Nature Reviews Neuroscience*.

[18] Matsumoto Y., Tsunekawa Y., Nomura T. (2011). Differential proliferation rhythm of neural progenitor and oligodendrocyte precursor cells in the young adult hippocampus. *PLoS ONE*.

[19] Ng F. S., Tangredi M. M., Jackson F. R. (2011). Glial cells physiologically modulate clock neurons and circadian behavior in a calcium-dependent manner. *Current Biology*.

[20] Hayashi Y., Koyanagi S., Kusunose N. (2013). The intrinsic microglial molecular clock controls synaptic strength via the circadian expression of cathepsin S. *Scientific Reports*.

[21] Hayashi Y., Koyanagi S., Kusunose N. (2013). Diurnal spatial rearrangement of microglial processes through the rhythmic expression of P2Y12 receptors. *Journal of Neurological Disorders*.

[22] Chi-Castañeda D., Waliszewski S. M., Zepeda R. C., Hernández-Kelly L. C. R., Caba M., Ortega A. (2015). Glutamate-dependent BMAL1 regulation in cultured Bergmann glia cells. *Neurochemical Research*.

[23] Fonken L. K., Frank M. G., Kitt M. M., Barrientos R. M., Watkins L. R., Maier S. F. (2015). Microglia inflammatory responses are controlled by an intrinsic circadian clock. *Brain, Behavior, and Immunity*.

[24] Danbolt N. C., Furness D. N., Zhou Y. (2016). Neuronal vs glial glutamate uptake: resolving the conundrum. *Neurochemistry International*.

[25] Halassa M. M., Florian C., Fellin T. (2009). Astrocytic modulation of sleep homeostasis and cognitive consequences of sleep loss. *Neuron*.

[26] Newman L. A., Korol D. L., Gold P. E. (2011). Lactate produced by glycogenolysis in astrocytes regulates memory processing. *PLoS One*.

[27] Suzuki A., Stern S. A., Bozdagi O. (2011). Astrocyte-neuron lactate transport is required for long-term memory formation. *Cell*.

[28] Han J., Kesner P., Metna-Laurent M. (2012). Acute cannabinoids impair working memory through astroglial CB1 receptor modulation of hippocampal LTD. *Cell*.

[29] Prolo L. M., Takahashi J. S., Herzog E. D. (2005). Circadian rhythm generation and entrainment in astrocytes. *Journal of Neuroscience*.

[30] Yagita K., Yamanaka I., Emoto N., Kawakami K., Shimada S. (2010). Real-time monitoring of circadian clock oscillations in primary cultures of mammalian cells using Tol2 transposon-mediated gene transfer strategy. *BMC Biotechnology*.

[31] Spanagel R., Pendyala G., Abarca C. (2005). The clock gene *Per2* influences the glutamatergic system and modulates alcohol consumption. *Nature Medicine*.

[32] Beaulé C., Swanstrom A., Leone M. J., Herzog E. D. (2009). Circadian modulation of gene expression, but not glutamate uptake, in mouse and rat cortical astrocytes. *PLoS One*.

[33] Beaulé C., Granados-Fuentes D., Marpegan L., Herzog E. D. (2011). *In vitro* circadian rhythms: imaging and electrophysiology. *Essays in Biochemistry*.

[34] Danbolt N. C. (2001). Glutamate uptake. *Progress in Neurobiology*.

[35] Brancaccio M., Patton A. P., Chesham J. E., Maywood E. S., Hastings M. H. (2017). Astrocytes control circadian timekeeping in the suprachiasmatic nucleus via glutamatergic signaling. *Neuron*.

[36] Tso C. F., Simon T., Greenlaw A. C., Puri T., Mieda M., Herzog E. D. (2017). Astrocytes regulate daily rhythms in the suprachiasmatic nucleus and behavior. *Current Biology*.

[37] Lavialle M., Servière J. (1993). Circadian fluctuations in GFAP distribution in the Syrian hamster suprachiasmatic nucleus. *Neuroreport*.

[38] Servière J., Lavialle M. (1996). Chapter 5 astrocytes in the mammalian circadian clock: putative roles. *Progress in Brain Research*.

[39] Girardet C., Becquet D., Blanchard M.-P., François-Bellan A. M., Bosler O. (2010). Neuroglial and synaptic rearrangements associated with photic entrainment of the circadian clock in the suprachiasmatic nucleus. *European Journal of Neuroscience*.

[40] Moriya T., Yoshinobu Y., Kouzu Y. (2000). Involvement of glial fibrillary acidic protein (GFAP) expressed in astroglial cells in circadian rhythm under constant lighting conditions in mice. *Journal of Neuroscience Research*.

[41] Shibuki K., Gomi H., Chen L. (1996). Deficient cerebellar long-term depression, impaired eyeblink conditioning, and normal motor coordination in GFAP mutant mice. *Neuron*.

[42] Slat E., Freeman G. M., Herzog E. D. (2013). The clock in the brain: neurons, glia, and networks in daily rhythms. *Handbook Experimental Pharmacology*.

[43] Leone M. J., Marpegan L., Bekinschtein T. A., Costas M. A., Golombek D. A. (2006). Suprachiasmatic astrocytes as an interface for immune-circadian signalling. *Journal of Neuroscience Research*.

[44] Jolly S., Journiac N., Vernet-der Garabedian B., Mariani J. (2012). RORalpha, a key to the development and functioning of the brain. *The Cerebellum*.

[45] Delerive P., Monté D., Dubois G. (2001). The orphan nuclear receptor RORα is a negative regulator of the inflammatory response. *EMBO Reports*.

[46] Journiac N., Jolly S., Jarvis C. (2009). The nuclear receptor RORα exerts a bi-directional regulation of IL-6 in resting and reactive astrocytes. *Proceedings of the National Academy of Sciences of the United States of America*.

[47] Morioka N., Tomori M., Zhang F. F., Saeki M., Hisaoka-Nakashima K., Nakata Y. (2016). Stimulation of nuclear receptor REV-ERBs regulates tumor necrosis factor-induced expression of proinflammatory molecules in C6 astroglial cells. *Biochemical and Biophysical Research Communications*.

[48] Ramakrishnan S. N., Lau P., Burke L. J., Muscat G. E. O. (2005). Reverb regulates the expression of genes involved in lipid absorption in skeletal muscle cells: evidence for cross-talk between orphan nuclear receptors and myokines. *Journal of Biological Chemistry*.

[49] Sato S., Sakurai T., Ogasawara J. (2014). Direct and indirect suppression of interleukin-6 gene expression in murine macrophages by nuclear orphan receptor REV-ERB*α*. *The Scientific World Journal*.

[50] Sato S., Sakurai T., Ogasawara J. (2014). A circadian clock gene, Rev-erbα, modulates the inflammatory function of macrophages through the negative regulation of *Ccl2* expression. *The Journal of Immunology*.

[51] Morioka N., Sugimoto T., Tokuhara M. (2012). Spinal astrocytes contribute to the circadian oscillation of glutamine synthase, cyclooxygenase-1 and clock genes in the lumbar spinal cord of mice. *Neurochemistry International*.

[52] Haydon P. G. (2001). GLIA: listening and talking to the synapse. *Nature Reviews Neuroscience*.

[53] Parpura V., Zorec R. (2010). Gliotransmission: exocytotic release from astrocytes. *Brain Research Reviews*.

[54] Womac A. D., Burkeen J. F., Neuendorff N., Earnest D. J., Zoran M. J. (2009). Circadian rhythms of extracellular ATP accumulation in suprachiasmatic nucleus cells and cultured astrocytes. *European Journal of Neuroscience*.

[55] Marpegan L., Swanstrom A. E., Chung K. (2011). Circadian regulation of ATP release in astrocytes. *Journal of Neuroscience*.

[56] Xu L., Ruan G., Dai H., Liu A. C., Penn J., McMahon D. G. (2016). Mammalian retinal Müller cells have circadian clock function. *Molecular Vision*.

[57] Tosini G., Menaker M. (1996). Circadian rhythms in cultured mammalian retina. *Science*.

[58] Nakazato R., Takarada T., Yamamoto T., Hotta S., Hinoi E., Yoneda Y. (2011). Selective upregulation of Per1 mRNA expression by ATP through activation of P2X7 purinergic receptors expressed in microglial cells. *Journal of Pharmacological Sciences*.

[59] Takayama F., Zhang X., Hayashi Y., Wu Z., Nakanishi H. (2017). Dysfunction in diurnal synaptic responses and social behavior abnormalities in cathepsin S-deficient mice. *Biochemical and Biophysical Research Communications*.

[60] Bhattacharjee Y. (2007). Is internal timing key to mental health?. *Science*.

[61] Hayashi Y., Wu Z., Nakanishi H. (2014). A possible link between microglial process dysfunction and neuropsychiatric disorders. *Journal of Neurological Disorders and Stroke*.

[62] Nakazato R., Hotta S., Yamada D. (2017). The intrinsic microglial clock system regulates interleukin-6 expression. *Glia*.

[63] Fünfschilling U., Supplie L. M., Mahad D. (2012). Glycolytic oligodendrocytes maintain myelin and long-term axonal integrity. *Nature*.

[64] McKenzie I. A., Ohayon D., Li H. (2014). Motor skill learning requires active central myelination. *Science*.

[65] Kochman L. J., Weber E. T., Fornal C. A., Jacobs B. L. (2006). Circadian variation in mouse hippocampal cell proliferation. *Neuroscience Letters*.

[66] Lee C. C. (2006). Tumor suppression by the mammalian period genes. *Cancer Causes and Control*.

[67] Chi-Castañeda D., Ortega A. (2016). Clock genes in glia cells: a rhythmic history. *ASN Neuro*.

[68] Yuferov V., Bart G., Kreek M. J. (2005). Clock reset for alcoholism. *Nature Medicine*.

[69] Domingues A. M., Taylor M., Fern R. (2010). Glia as transmitter sources and sensors in health and disease. *Neurochemistry International*.

[70] Yuferov V., Kroslak T., Laforge K. S., Zhou Y., Ho A., Kreek M. J. (2003). Differential gene expression in the rat caudate putamen after binge cocaine administration: advantage of triplicate microarray analysis. *Synapse*.

[71] Aston C., Jiang L., Sokolov B. P. (2004). Microarray analysis of postmortem temporal cortex from patients with schizophrenia. *Journal of Neuroscience Research*.

[72] Nievergelt C. M., Kripke D. F., Barrett T. B. (2006). Suggestive evidence for association of the circadian genes *PERIOD3* and *ARNTL* with bipolar disorder. *American Journal of Medical Genetics Part B: Neuropsychiatric Genetics*.

[73] Benedetti F., Dallaspezia S., Colombo C., Pirovano A., Marino E., Smeraldi E. (2008). A length polymorphism in the circadian clock gene Per3 influences age at onset of bipolar disorder. *Neuroscience Letters*.

[74] Krishnan N., Kretzschmar D., Rakshit K., Chow E., Giebultowicz J. M. (2009). The circadian clock gene *period* extends healthspan in aging *Drosophila melanogaster*. *Aging*.

[75] Gerstner J. R. (2010). The aging clock: to ‘BMAL’icious toward learning and memory. *Aging*.

[76] Gu Z., Wang B. B., Zhang Y. B. (2015). Association of *ARNTL* and *PER1* genes with Parkinson’s disease: a case-control study of Han Chinese. *Scientific Reports*.

[77] Song H., Moon M., Choe H. K. (2015). Aβ-induced degradation of BMAL1 and CBP leads to circadian rhythm disruption in Alzheimer’s disease. *Molecular Neurodegeneration*.

[78] Andretic R., Chaney S., Hirsh J. (1999). Requirement of circadian genes for cocaine sensitization in Drosophila. *Science*.

[79] Niculescu A. B., Segal D. S., Kuczenski R., Barrett T., Hauger R. L., Kelsoe J. R. (2000). Identifying a series of candidate genes for mania and psychosis: a convergent functional genomics approach. *Physiological Genomics*.

[80] Bhat R. V., Budd S. L. (2002). GSK3β signalling: casting a wide net in Alzheimer’s disease. *Neuro-Signals*.

[81] Soria V., Martínez-Amorós E., Escaramís G. (2010). Differential association of circadian genes with mood disorders: CRY1 and NPAS2 are associated with unipolar major depression and CLOCK and VIP with bipolar disorder. *Neuropsychopharmacology*.

[82] Geoffroy P. A., Lajnef M., Bellivier F. (2015). Genetic association study of circadian genes with seasonal pattern in bipolar disorders. *Scientific Reports*.

[83] Naylor E., Bergmann B. M., Krauski K. (2000). The circadian *clock* mutation alters sleep homeostasis in the mouse. *The Journal of Neuroscience*.

[84] Wisor J. P., O'Hara B. F., Terao A. (2002). A role for cryptochromes in sleep regulation. *BMC Neuroscience*.

[85] Laposky A., Easton A., Dugovic C., Walisser J., Bradfield C., Turek F. (2005). Deletion of the mammalian circadian clock gene *BMAL1/Mop3* alters baseline sleep architecture and the response to sleep deprivation. *Sleep*.

[86] Irwin M., McClintick J., Costlow C., Fortner M., White J., Gillin J. C. (1996). Partial night sleep deprivation reduces natural killer and cellular immune responses in humans. *FASEB Journal*.

[87] Born J., Lange T., Hansen K., Mölle M., Fehm H. L. (1997). Effects of sleep and circadian rhythm on human circulating immune cells. *The Journal of Immunology*.

[88] Lam M. T. Y., Cho H., Lesch H. P. (2013). Rev-Erbs repress macrophage gene expression by inhibiting enhancer-directed transcription. *Nature*.

[89] Nguyen K. D., Fentress S. J., Qiu Y., Yun K., Cox J. S., Chawla A. (2013). Circadian gene *Bmal1* regulates diurnal oscillations of Ly6C^hi^ inflammatory monocytes. *Science*.

[90] Castanon-Cervantes O., Wu M., Ehlen J. C. (2010). Dysregulation of inflammatory responses by chronic circadian disruption. *Journal of Immunology*.

[91] Fonken L. K., Weil Z. M., Nelson R. J. (2013). Mice exposed to dim light at night exaggerate inflammatory responses to lipopolysaccharide. *Brain, Behavior, and Immunity*.

[92] Saijo K., Glass C. K. (2011). Microglial cell origin and phenotypes in health and disease. *Nature Reviews Immunology*.

[93] Louboutin J.-P., Strayer D. S. (2013). Relationship between the chemokine receptor CCR5 and microglia in neurological disorders: consequences of targeting CCR5 on neuroinflammation, neuronal death and regeneration in a model of epilepsy. *CNS and Neurological Disorders Drug Targets*.

[94] Nakagawa Y., Chiba K. (2015). Diversity and plasticity of microglial cells in psychiatric and neurological disorders. *Pharmacology and Therapeutics*.

